# Genome-wide association study, genomic prediction and marker-assisted selection for seed weight in soybean (*Glycinemax*)

**DOI:** 10.1007/s00122-015-2614-x

**Published:** 2015-10-30

**Authors:** Jiaoping Zhang, Qijian Song, Perry B. Cregan, Guo-Liang Jiang

**Affiliations:** Plant Science Department, South Dakota State University, Brookings, SD 57006 USA; Soybean Genomics and Improvement Laboratory, US Department of Agriculture, Agricultural Research Service (USDA-ARS), 10300 Baltimore Ave, Beltsville, MD 20705 USA; Agricultural Research Station, Virginia State University, Carter G. Woodson Ave, P.O. Box 9061, Petersburg, VA 23806 USA

## Abstract

*****Key message***:**

**Twenty-two loci for soybean SW and candidate genes conditioning seed development were identified; and prediction accuracies of GS and MAS were estimated through cross-validation and validation with unrelated populations.**

**Abstract:**

Soybean (*Glycine max*) is a major crop for plant protein and oil production, and seed weight (SW) is important for yield and quality in food/vegetable uses of soybean. However, our knowledge of genes controlling SW remains limited. To better understand the molecular mechanism underlying the trait and explore marker-based breeding approaches, we conducted a genome-wide association study in a population of 309 soybean germplasm accessions using 31,045 single nucleotide polymorphisms (SNPs), and estimated the prediction accuracy of genomic selection (GS) and marker-assisted selection (MAS) for SW. Twenty-two loci of minor effect associated with SW were identified, including hotspots on Gm04 and Gm19. The mixed model containing these loci explained 83.4 % of phenotypic variation. Candidate genes with *Arabidopsis* orthologs conditioning SW were also proposed. The prediction accuracies of GS and MAS by cross-validation were 0.75–0.87 and 0.62–0.75, respectively, depending on the number of SNPs used and the size of training population. GS also outperformed MAS when the validation was performed using unrelated panels across a wide range of maturities, with an average prediction accuracy of 0.74 versus 0.53. This study convincingly demonstrated that soybean SW is controlled by numerous minor-effect loci. It greatly enhances our understanding of the genetic basis of SW in soybean and facilitates the identification of genes controlling the trait. It also suggests that GS holds promise for accelerating soybean breeding progress. The results are helpful for genetic improvement and genomic prediction of yield in soybean.

**Electronic supplementary material:**

The online version of this article (doi:10.1007/s00122-015-2614-x) contains supplementary material, which is available to authorized users.

## Introduction

Soybean (*Glycine max*), rich in both protein and oil, is one of the most economically important crops. It accounted for approximately 68 % of world protein meal consumption and 57 % of world oilseed production during the past decades (USDA-FAS, http://www.fas.usda.gov/). As a yield component, seed weight (SW) is a complex and agronomically important trait in soybean. It is considerably attributed to seed size, which is an important character of soybean cultivars and affects the quality of many soy products for human consumption, such as soy sprouts, soy nuts, edamame, soy sauce, natto and miso (Clarke and Wiseman 2000; Friedman and Brandon 2001). Therefore, dissecting the genetic basis of SW is helpful to improve soybean yield potential and soy food as well.

Seeds of flowering plants consist of three major compartments: embryo, endosperm and seed coat. Embryo and endosperm are two zygotic products of double fertilization with different genotypes. The embryo, that gives rise to the daughter plant, is diploid with a copy of genome from each parent. Whereas the endosperm, that provides nutrition for embryogenesis and seed germination, is triploid with one and two genome equivalents from paternal and maternal parent, respectively (Lopes and Larkins 1993). By contrast, the seed coat that encloses the embryo and endosperm is strictly of maternal origin and differentiated from maternal-derived integuments (Haughn and Chaudhury 2005). It can be further divided into an inner and outer coat known as the tegmen and testa, respectively. The final seed size and weight is determined by the coordination among the embryo, endosperm and seed coat. In dicots like soybean, however, the endosperm is absorbed by the cotyledon, a part of embryo.

Research in *Arabidopsis* suggested that the cell division and elongation of embryo, endosperm and integument, which are predominantly regulated by phytohormones, determine the final size of seeds (Jiang et al. 2013; Ohto et al. 2005; Riefler et al. 2006; Schruff et al. 2006; Singh et al. 2002). The *Arabidopsis* AUXIN RESPONSE FACTOR 2 (ARF2), one of the ARF transcription factor family members that bind to auxin-responsive elements in the promoters of auxin-regulated genes, plays a central role in various auxin-mediated developmental processes including seed growing (Okushima et al. 2005). The loss-of-function mutant *arf2* resulted in an increased seed size and weight due to extra cell division in the integuments (Schruff et al. 2006). The *Arabidopsis* histidine kinase 2 (AHK2), AHK3 and AHK4 (also known as cytokinin receptors) fulfill important roles in cytokinin-dependent endospermal and maternal control of embryo size. Triple mutant *ahk2,3,4* showed increased cell number and cell size of the embryo, resulting in enlarged seed size (Riefler et al. 2006). The transcriptional factor *Arabidopsis* APETALA2 (AP2), an ethylene responsive element binding protein and functioning in the floral and seed development (Jofuku et al. 1994), has broad effects on the development of embryo, endosperm and seed coat. Large seed size was observed in loss-of-function mutant *ap2*, which exhibited elongated integument cells, enlarged embryo and endosperm (Ohto et al. 2005).

In soybean, research has shown that seed size and weight are controlled by both environmental and genetic factors. The temperature and water availability during seed filling and the duration of seed filling substantially affect seed size and weight (Dornbos Jr and Mullen 1991; Egli et al. 1978). Genetic studies have identified many quantitative trait loci (QTLs) associated with seed weight or related traits in soybean (SoyBase, http://www.soybase.org/). However, because of the limited recombination or insufficient maker density, the previously identified chromosomal regions associated with SW were usually not fine enough to access candidate gene(s) (Niu et al. 2013; Salvi and Tuberosa 2005). This also hinders breeding efforts to improve seed weight and size in soybean through marker-assisted selection (MAS). Seed size in soybean is also believed to be relevant to domestication (Liu et al. 2007). The cultivated soybeans have larger seed size and weight than their wild relatives (*G. soja*). A recent study indicated that the *GmCYP78A10* gene, strongly associated with seed size in soybean, underwent artificial selection during early stage of soybean domestication (Wang et al. 2014). However, the molecular mechanism underlying SW and seed size in soybean remains unclear although significant advance has been achieved.

With the decreased genotyping cost and improved statistical methods, genome-wide association study (GWAS) and genomic selection (GS) present promising prospects for genetic improvement of complex traits in crop species. GWAS with a population of unrelated lines and high-density single nucleotide polymorphism (SNP) markers is capable of identifying causal genes for a broad range of complex traits in different crops (Huang et al. 2010; Li et al. 2013; Morris et al. 2013). In soybean, it has been used to disclose the genetic architecture of agronomic traits (Zhang et al. 2015), seed composition (Hwang et al. 2014) and disease resistance (Wen et al. 2014). GS refers to marker-based selection by capturing the total genetic variance with genome-wide markers without identifying a subset of trait-associated markers (Meuwissen et al. 2001). In general, GS is expected to be more effective than MAS that based on a few loci for quantitatively inherited traits (Bernardo and Yu 2007). A large number of GS studies have been reported in crop species such as maize (Albrecht et al. 2011; Bernardo 1996; Piepho 2009; Technow et al. 2013) and wheat (Heffner et al. 2011; Poland et al. 2012; Rutkoski et al. 2011) for various agronomic traits and disease resistance. However, its application to soybean is rarely addressed (Bao et al. 2014; Jarquin et al. 2014), and validation with unrelated population has not been reported.

Therefore, the objectives of this study were to (1) better understand the genetic architecture underlying SW in soybean, and (2) explore the potential of marker-based prediction as a new approach in soybean breeding. Twenty-two loci associated with SW and putative candidate genes with *Arabidopsis* orthologs involved in seed mass determination were identified via GWAS. GS exhibited higher prediction accuracies than MAS in all the tests of both cross-validation and validation with unrelated panels obtained from GRIN (http://www.ars-grin.gov/). This study enhances our understanding of the genetic architecture of SW and expedites the identification of genes conditioning SW in soybean. It demonstrates that GS can increase breeding efficiency and is also useful for genomic prediction of yield in soybean.

## Materials and methods

### Plant materials and field trials

Three hundred and nine plant introductions (PIs), obtained from the USDA Soybean Germplasm Collection, were planted in single-row plots in a randomized complete block design with three replications at four environments: Aurora (44°18′N and 96°40′W, 2011), Brookings (44°27′N and 96°47′W, 2010 and 2012) and Watertown (45°06′N and 97°05′W, 2012) in South Dakota, USA. The plots were 3.05 m in length and 0.76 m in width (or row spacing), and 86 seeds were sown per plot/row. According to the GRIN (http://www.ars-grin.gov/), 92 % of the PIs are maturity group (MG) 0 and 8 % MG 00, and 91 % originated from Northern China. The detailed information about the 309 PIs was given in our previous publication (Zhang et al. 2015).

### Phenotypic evaluation and statistical analysis

All plots were bulk-harvested individually after full maturity (R8 stage), and the seeds were dried in an air dryer. A sample of 100 cleaned seeds from each plot was randomly taken and weighed, and the data were presented as grams per 100-seed. The model for the phenotypic trait was *y*_*ijk*_ = *µ* + *g*_*i*_ + *l*_*j*_ + *(gl)*_*ij*_ + *b*_*k(j)*_ + *e*_*ijk*_, where *µ* is the overall mean, *g*_*i*_ is the genetic effect of the *i*th genotype, *l*_*j*_ is the effect of the *j*th environment, (*gl*)_*ij*_ is the interaction effect between the *i*th genotype and the *j*th environment, *b*_*k*(*j*)_ is the block effect within the *j*th environment, and *e*_*ijk*_ is a random error following *N*(0, *σ*_*e*_^2^). Broad-sense heritability was calculated on an entry-mean basis as *H*^2^ = *σ*_*g*_^2^/[*σ*_*g*_^2^ + σ_*gl*_^2^/*k* + σ_*e*_^2^/(*rk*)], where *σ*_*g*_^2^ is the genotypic variance, *σ*_*gl*_^2^ is the genotype by environment interaction variance, *k* is the number of environments, *r* is the number of replications. Estimation of variance components was performed by the varcomp procedure in SAS version 9.3 (SAS Institute, Inc., Cary, NC), with all effects considered to be random. The likelihood-ratio-based *R*^2^ was calculated to estimate the proportion of total variation explained by the mixed linear model (MLM) containing all identified loci for trait as described by Sun et al. (2010).

### Genotyping and quality control

The Illumina Infinium SoySNP50K BeadChip was used to genotype the population as described by Song et al. (2013), and 42,509 SNPs were identified with a call success rate of 85 % or greater. Of them, 61 SNPs present in unanchored sequence scaffolds were excluded from further analysis. The dataset had a missing rate of 0.6 %, and the missing data were imputed using BEAGLE version 3.3.1 with default parameter settings (Browning and Browning 2007, 2009). SNPs with a MAF < 5 % in the population were excluded from further analysis as well. Finally, a total of 31,045 SNPs were used for GWAS. The genotypic data of these SNPs for the four GRIN panels were downloaded from the SoyBase (http://www.soybase.org/snps/index.php), and the imputation analysis was performed same as described above for the main association panel.

### Linkage disequilibrium estimation

Pairwise LD between markers was calculated as squared correlation coefficient (*r*^2^) of alleles using R package synbreed (Wimmer et al. 2012). Due to the substantial difference in recombination rate between euchromatic and heterochromatic regions, *r*^2^ was calculated separately for the two chromosomal regions. The physical length of euchromatic and heterochromatic regions for each chromosome was defined as in the Gmax1.01 reference genome (SoyBase, www.soybase.org). Only *r*^2^ for SNPs with pairwise distance less than 10 Mb in either euchromatic or heterochromatic regions was used to draw the average LD decay figure by R script using the equation described by Remington et al. (2001). The LD decay rate of the population was measured as the chromosomal distance where the average *r*^2^ dropped to half its maximum value (Huang et al. 2010).

### Genome-wide association analysis

To minimize the effects of environmental variation, best linear unbiased predictions (BLUPs) of genetic effect for each line were calculated using the R package lme4 (Bates et al. 2012) in the same model as described for phenotypic trait. The BLUPs were then used to fit various models for association analysis. The one-way ANOVA model for naive test, without correction of population structure and familial relatedness, was implemented in R (Team 2012). General linear model (GLM) with population structure and MLM accounting for both population structure and kinship were implemented in the Genomic Association and Prediction Integrated Tool (GAPIT) R package (Lipka et al. 2012; Zhang et al. 2010).

For the naive test, the equation was

$$y = \mu + X\alpha + e.$$

For the GLM analysis, the equation was

$$y = \mu + X\alpha + P\beta + e.$$

For the MLM analysis, the equation was

$$y = \mu + X\alpha + P\beta + Zu + e,$$

where *y* is a *N* × 1 vector of BLUPs of genetic effect (*N* is the number of line), *μ* is the overall mean, *X* is the incidence matrix relating the individuals to the fixed marker effects *α*, *P* is the incidence matrix relating the individuals to the fixed principal component (PC) effects *β*, and *Z* is the incidence matrix relating the individuals to the random group effects *u* obtained from the compression algorithm. The random group effects *u* follows a multivariate normal distribution with mean 0 and variance–covariance matrix 2*KV*_*g*_, where *K* is the kinship matrix, and *V*_*g*_ is the genetic variance component. The random error term *e* follows a multivariate normal distribution with mean 0 and variance–covariance matrix *IV*_*e*_, where *I* is the identity matrix and *V*_*e*_ is the error variance component. GLM, compressed MLM and regular MLM could be achieved by set different level of compression as described by the manual of GAPIT. The first four PCs were involved in models as covariates according to Bayesian Information Criterion (BIC) test of the model fitness. The significance threshold for SNP-trait associations was determined by false discovery rate (*q* value) <0.05 or *P* < 7.9 × 10^−5^.

### Genomic prediction and marker-assisted selection

Both cross-validation and validation with unrelated panels were conducted for GS and MAS. A modified cross-validation was performed as described by Wurschum et al. (2013) to estimate the prediction accuracy. Briefly, for a fivefold cross-validation, 62 PIs (20 %) of the association panel were randomly assigned to a validation set for each prediction, and all of the remaining PIs (247, 80 % of the association panel) were used as the training set. For the training population size-effect analysis, a subgroup (97, 147 or 197) of the remains was randomly selected as the training set. The loci effects were then estimated based on genotypic and phenotypic data of the training set. Finally, the estimates of loci effect were used to predict the genomic estimated breeding values (GEBVs) of the validation set based on the genotypic data. The whole process was repeated for 1000 times. Ridge regression best linear unbiased prediction (RR- BLUP) was used to predict GEBVs in GS, as it has been demonstrated an effective prediction method with high accuracy across a wide range of traits and crops (Heslot et al. 2012; Jarquin et al. 2014; Lipka et al. 2014). While in MAS, multiple linear regression (MLR) was employed. The prediction accuracy was calculated by dividing the average Pearson’s correlation (*r*) between the BLUPs of genetic effects and the GEBVs with the square root of the heritability (Resende et al. 2012). To investigate the prediction accuracies with different number of markers, three sets of markers (2000, 1000 and 500 SNPs in total, respectively) were formed by randomly selecting 100, 50 or 25 SNPs from each of the 20 chromosomes. Each set of markers was then used in the cross-validation as described above.

The RR-BLUP approach was conducted using the rrBLUP package (Endelman 2011) implemented in R (Team 2012). Briefly, the following model was used for estimation of the marker effect in the training set:

$$y = \mu + X\beta + e,$$

where *y* is a *t* × 1 vector of BLUPs of genetic effect (t is the size of the training set), *β* is a *m* × 1 vector of marker effect and assumed random effect with Var[*β*] = *Kσ*_*β*_^2^, where *m* is the number of marker and *K* is the identity matrix (Endelman 2011), *X* is the marker genotype matrix, and *e* is the residual error. The GEBVs of the validation set were predicted as:

$$\hat{y} = \mu + X\hat{\beta }.$$

For MAS by MLR method, different numbers of loci were selected from the 22 loci identified via GWAS through stepwise algorithm based upon Akaike information criterion (AIC), which was implemented using R package MASS (Venables and Ripley 2002). While a control test was conducted typically for MAS with 15 loci, which gives the highest prediction accuracy in cross-validations. Briefly, 15 markers were randomly selected from the 31,045 SNPs without replacement. The random sampling followed by cross-validations was repeated 1000 times. The marker effects of the training set were estimated using the fixed effects model:

$$y = \mu + X_{1} \beta_{1} + \cdots + X_{i} \beta_{i} + e,$$

where *y* is the vector of BLUPs of genetic effect (same as used for GWAS), *μ* is the mean, *β*_*i*_ is the fixed effect of the selected marker *i*, *X*_*i*_ is the marker genotype matrix of selected marker *i*, and *e* is the residual error. The GEBV of each individual *j* in validation set based on the selected markers was calculated as:

$$\hat{y}_{j} = \mu + X_{1} \hat{\beta }_{1} + \cdots + X_{l} \hat{\beta }_{l} ,$$

in which *l* is the number of selected marker.

Both GS and MAS were validated with four unrelated data sets taken from GRIN (http://www.ars-grin.gov/cgi-bin/npgs/html/desc.pl?51015). They were SOYBEAN.EVALUATION.1MN63 (MN63), SOYBEAN.EVALUATION.1IL64 (IL64), SOYBEAN.EVALUATION.3IL83.2 (IL83.2) and SOYBEAN.EVALUATION.MS989 (MS989) (Supplementary file Table S1). Lines presented in the association panel were excluded from analyses, and thus the final population size was 270, 724, 192 and 425 for MN63, IL64, IL83.2 and MS989, respectively. The whole population of each panel was used for validation based on the marker effects estimated from the association panel. The prediction accuracies for these validations were simply estimated as the Pearson’s correlation (*r*), as no heritability was available for adjustment.

## Results

### Statistics of phenotypes

The averaged SW over four environments showed a continuous distribution in the GWAS panel of 309 soybean PIs, with a wide range of variation from 7.3 to 23.6 g per 100-seed (Supplementary file Fig. S1). The ANOVA indicated that effects of genotypes, environments and their interaction were significant (Supplementary file Table S2). The correlations of trait performance across environments were quite high, averaged *r* > 0.85 (*P* < 10^−10^), indicating high repeatability of the trait performance. The estimate of broad-sense heritability was 0.97, suggesting that the majority of phenotypic variation in soybean SW is attributed to genetic effects.

### Distribution of markers and linkage disequilibrium

A total of 31,045 SNPs with minor allele frequency (MAF) ≥0.05 was used to GWAS for SW after quality control, resulting in a genome-wide marker density of 29 kb per SNP. On the SoySNP50K, however, markers were designed to unevenly distribute across chromosomal regions due to the substantial difference in recombination ratio between euchromatic and heterochromatic regions (Song et al. 2013). In the present study, 74.6 % of SNPs were located in euchromatic regions, exhibiting a marker density of 20 kb per SNP in euchromatic region but 62 kb per SNP in heterochromatic region. Accordingly, the linkage disequilibrium (LD) decayed (*r*^2^ drop to half of its maximum value) at 326 kb in euchromatic region, while in heterochromatic region LD did not decay until 4285 kb (Supplementary file Fig. S2).

### Genome-wide association analysis of seed weight

The MLM, taking both population structure and relative kinship into account (Yu et al. 2006; Zhang et al. 2010), was employed to conduct the association analysis. Principal component analysis was performed with the whole set SNPs to capture the overall population stratification of the association panel. The first four PCs that explained 28.2 % of the total genetic variation were involved in the MLM according to the BIC test (Supplementary file Table S3). The first three PCs were presented in the supplementary file Fig. S3. Compared with the GLM, which involves population structure only, and the naive model, which involves neither population structure nor individual relationship, the MLM showed a greater control of genomic inflation (type I error) (Supplementary file Fig. S4). We also conducted GWAS with a compressed MLM. However, similar results were found between the regular and the compressed MLM (Supplementary file Fig. S4). Therefore, all further analysis and results presented were referred to GWAS using the regular MLM.

**Fig. 1:**
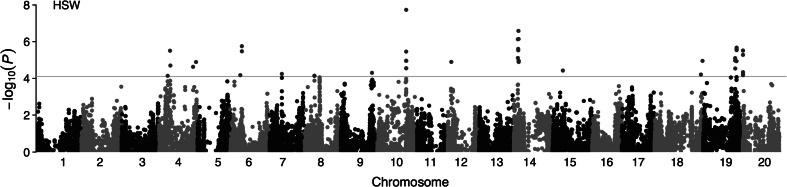
Manhattan plot of GWAS for 100-seed weight (HSW) in soybean. Negative log_10_-transformed *P* values of SNPs from a genome-wide scan for HSW using mixed linear model including both kinship and populations structure are plotted against positions on each of the 20 chromosomes. The significant traits-associated SNPs (*P* < 7.9 × 10^−5^) are distinguished by the *threshold line* and colored in *red* (color figure online)

Through GWAS, 48 SNPs significantly associated with SW were identified across 12 of 20 soybean chromosomes (Fig. 1). The contribution of a single SNP to the phenotypic variation was 1.8–3.8 % under MLM circumstance. For convenience of further analysis, the significant trait-associated SNPs located in close proximity were clumped at LD *r*^2^ > 0.70 and the lead SNP was used to represent the locus (Table 1). As a result, 22 loci associated with SW were identified. Eight of them included multiple SNPs with the distance ranging from 14.7 to 427.6 kb between markers. The MLM containing all the 22 loci explained 83.4 % of phenotypic variation, suggesting that additive effects predominantly condition SW in soybean and pyramiding of desired alleles can be an effective way to improve soybean SW.

**Table 1:** Loci and SNPs significantly associated with seed weight, predicted candidate genes and previously reported QTLs for seed weight at similar genome regions

Loci	SNP ID^a^	Alleles^b^	MAF	Allelic effect	R^2^	*P* value	Known QTLs^c^	Candidate genes^d^	Annotation
SW1	Gm04_11182315_A_G	G:A	0.36	0.54	0.018	7.27E−05	Seed weight 5-2		
SW2	Gm04_14213918_C_T	T:C	0.29	0.73	0.026	3.11E−06	Seed weight 20-2, 36-15, seed length 1-14, seed height 1-13 and seed volume 1-10		
SW3	Gm04_43653965_T_C	T:C	0.12	0.73	0.021	2.35E−05			
SW4	Gm04_47414790_G_A	A:G	0.10	0.81	0.022	1.28E−05			
SW5	Gm06_13151347_C_T	C:T	0.08	0.89	0.018	6.71E−05			
SW6	Gm06_15115808_C_T	T:C	0.13	0.70	0.027	1.76E−06	Seed weight 2-2		
SW7	Gm06_15154965_T_C	C:T	0.05	0.97	0.025	3.34E−06	Seed weight 2-2		
SW8	Gm07_15662403_C_T	T:C	0.34	−0.43	0.019	5.71E−05			
SW9	Gm08_13444545_A_G	G:A	0.06	0.75	0.018	7.26E−05	Seed weight 34-13 and 35-1		
SW10	Gm09_40164588_A_G	G:A	0.10	0.72	0.019	4.98E−05	Seed weight 2-7 and 27-3		
SW11	Gm10_37088544_G_T	G:T	0.06	1.29	0.038	1.84E−08	Seed weight 27-2, 34-8, 25-4 and 37-6	Glyma10g28250	Transcription factor MYB61-like
SW12	Gm12_4670638_A_C	C:A	0.06	0.91	0.022	1.27E−05			
SW13	Gm14_5352488_T_G	G:T	0.08	0.78	0.029	7.35E−07	Seed weight 36-14		
SW14	Gm14_6072858_G_A	A:G	0.12	0.72	0.031	2.58E−07	Seed weight 36-14	Glyma14g08050	ARM repeat superfamily protein
SW15	Gm14_6324126_G_A	A:G	0.06	1.29	0.029	7.16E−07	Seed weight 36-14	Glyma14g08280	Nucleotide-diphospho-sugar transferase family protein
SW16	Gm15_13314408_G_A	A:G	0.05	0.86	0.020	3.75E−05	Seed weight 29-2		
SW17	Gm18_59490788_A_G	G:A	0.06	0.71	0.019	6.18E−05			
SW18	Gm18_61540919_C_T	T:C	0.08	0.89	0.023	1.11E−05			
SW19	Gm19_41013395_C_T	C:T	0.27	0.54	0.023	7.88E−06	Seed weight 35-7, 15-7	Glyma19g33421	AHP protein
SW20	Gm19_41144271_A_G	A:G	0.05	0.98	0.025	2.89E−05	Seed weight 35-7, 15-7	Glyma19g33550	Unknown
SW21	Gm19_42921997_A_G	G:A	0.43	−0.53	0.026	2.11E−06	Seed weight 5-1, 15-7, 17-1, 34-7, 35-7, 36-7. Seed volume 1-7, 1-8, seed length 1-10 and seed height 1-10,1-11, seed width 1-8	Glyma19g35180	AUX/IAA family protein
SW22	Gm20_481573_G_A	A:G	0.35	−0.49	0.026	3.06E−06	Seed weight 34-5		

^a^Start with the version of Joint Genome Institute (JGI 1.01) *G.max* genome sequence followed by chromosome number, physical position of the marker on that chromosome and two alleles of the locus (Schmutz et al. 2010). The first of the two alleles for each locus is the Williams 82 alleles

^b^Respect to minor allele

^c^Based on the QTL list on SoyBase (http://www.soybase.org)

^d^Genes annotated in Glyma1.1, Glyma1.0, and NCBI RefSeq gene models in SoyBase (http://www.soybase.org) were used as the source of candidate genes

### Loci effects and prediction of candidate genes

**Fig. 2:**
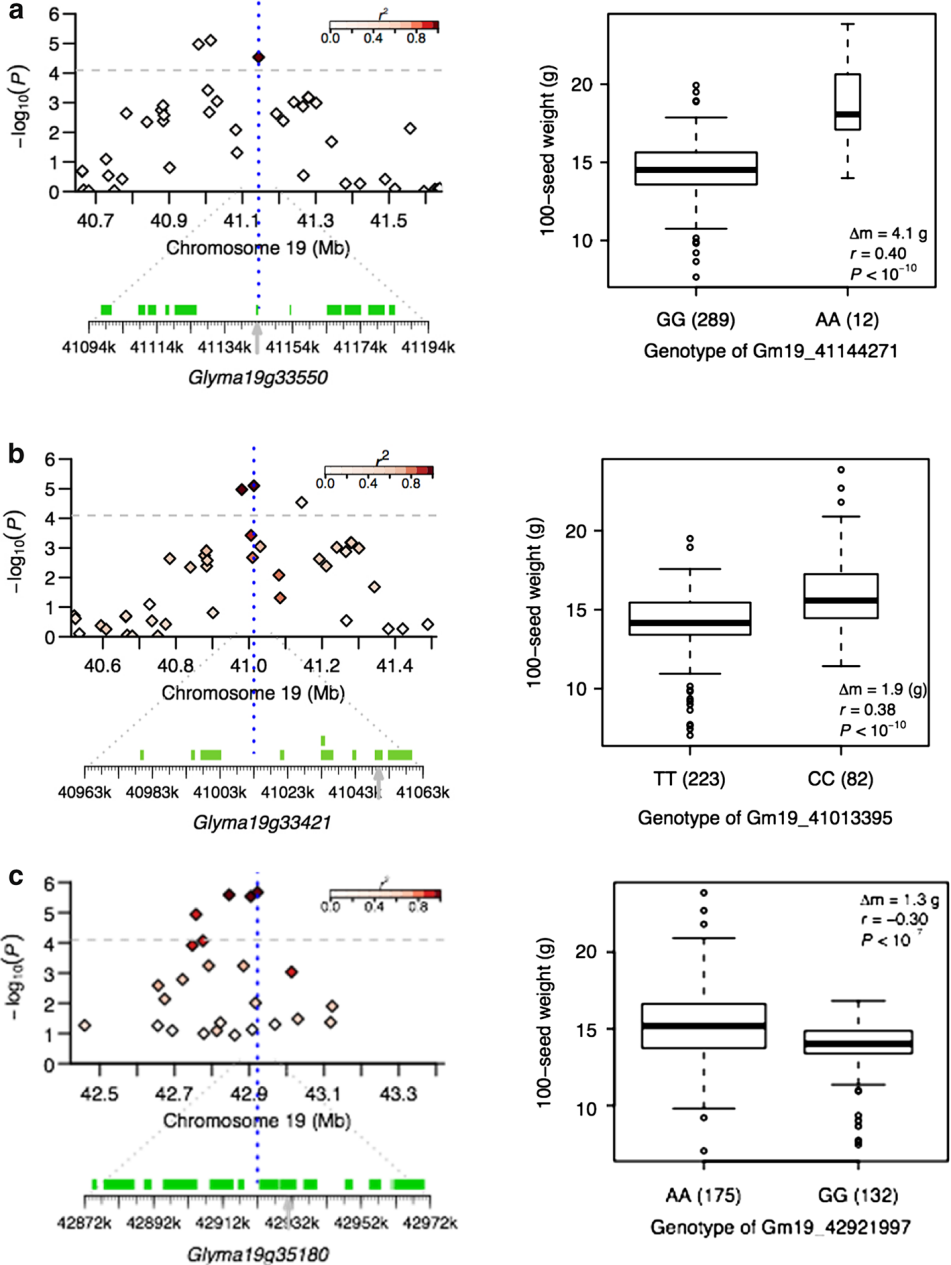
Candidate genes for loci associated with seed weight on Gm19 and phenotypic difference between different alleles of each locus. **a** SW20, **b** SW19 and **c** SW21. *Top of the left panel* shows a 0.5-Mb region each side of the lead SNP, whose position is indicated by a *vertical blue dashed line*. Negative log_10_-transformed *P* values of SNPs from the MLM are plotted on the *vertical axis*. Significant threshold is indicated as the *gray dashed line* at *q* = 0.05. The *color of each SNP* indicates its *r* ^2^ value with the lead SNP as shown in the *color index on the right top of the panel*. *Bottom of the left panel* shows putative genes within 50 kb adjacent region each side of the lead SNP as indicated by *green bars*. Candidate gene is indicated by *arrow*. The *boxplot on the right* shows the distribution of average 100-seed weight over four environments for each locus allele. The number of individual for each allele is given in the *parenthesis*. The *box* shows the first, second (median) and third quartile. The width of the box is proportional to the square root of the number of individuals for each allele. The whiskers extend to the 1.5 times of interquartile or the data extreme whichever is smaller. The difference of mean (Δ*m*), correlation coefficient (*r*) and *P* value for the correlation is also given (color figure online)

Three loci associated with seed weight, SW19, SW20 and SW21, were identified on Gm19. SW20 was target by the SNP Gm19_41144271_A_G (MAF = 0.05). It was located 336 bp upstream of the transcript start site of a putative gene, *Glyma19g33550*. There was an average difference of 4.1 g per 100-seed between two alleles of this locus (Fig. 2a). SW19 and SW21 were located 130.8 kb upstream and 1613.3 kb downstream of SW20, respectively. The two SNPs involved in SW19 were in complete LD (*r*^2^ = 1) and were 33.1 kb apart each other. The putative gene *Glyma19g33421*, identified close to the lead SNP Gm19_41013395_C_T (MAF = 0.27) (Fig. 2b), encodes a protein sharing 77.1 % similarity with the *Arabidopsis* HISTIDINE-CONTAINING PHOSPHOTRANSFER FACTOR 5 (AHP5) (Phytozome, http://www.phytozome.net). On average, the individuals carrying the minor frequency allele of the lead SNP exhibited 1.9 g higher than those with major frequency allele in 100-seed weight (Fig. 2b). SW21 was led by the SNP Gm19_42921997_A_G at the 42.9 Mb position on Gm19 with a high MAF of 0.43 (Fig. 2c). A putative gene, *Glyma19g35180*, encoding an AUX/IAA family protein, was found at 6.5 kb away from the lead SNP. The 100-seed weight of individuals with minor frequency allele at this locus was 1.3 g lower than those with major frequency allele (Fig. 2c).

**Fig. 3:**
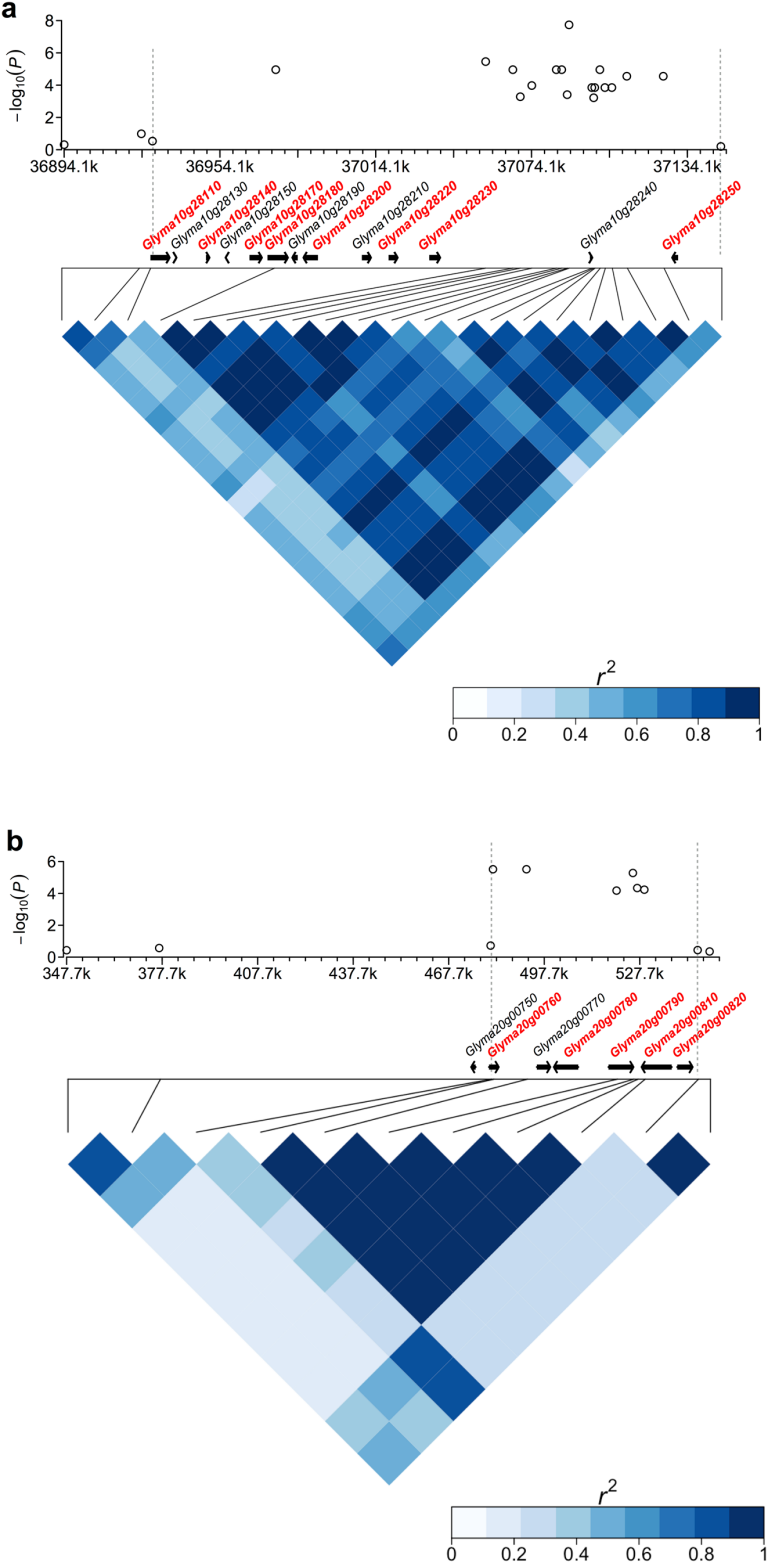
Candidate genome ranges for seed weight loci SW11 and SW22. Shown are genome regions harboring SW11 on Gm10 (**a**) and SW22 on Gm20 (**b**). In the *top panel*, the negative log_10_-transformed *P* values of SNPs from GWAS for seed weight are plotted against the physical positions of the given chromosomal region. The *bottom panel* depicts the extent of LD in this region on *r* ^2^. The *r* ^2^ values are indicated with *color key*. The candidate region for the locus was indicated by two vertical *dashed lines* in *gray*. Genes within this region are indicated in the *middle panel*. Those with transcript accumulated during seed filling were highlighted in *red* and *bold* according to the “seed development transcript count” track on SoyBase (http://soybase.org/) (color figure online)

The locus SW11, which was located at the 37.1 Mb position on Gm10, represented the strongest association in the present study with an allelic effect of 1.29 g per 100-seed. LD analysis revealed that a block about 218 kb length was related to this locus (Fig. 3a). Thirteen putative genes reside in this region. Among them, a MYB61-like transcription factor, encoded by the putative gene *Glyma10g28250*, was found at 35 kb downstream of the lead SNP of SW11 (Gm10_37088544_G_T, MAF = 0.06) (Fig. 3a). Another locus, SW22, was located at the 0.48 Mb position on Gm20. It was targeted by six SNPs with an average MAF larger than 0.35, indicating the high reliability of the marker-trait association. These SNPs were in high LD (*r*^2^ > 0.90) and corresponded to an LD block with about 65 kb in length on Gm20. There were six putative genes located in this region (Fig. 3b).

### Genomic prediction and MAS for seed weight

**Fig. 4:**
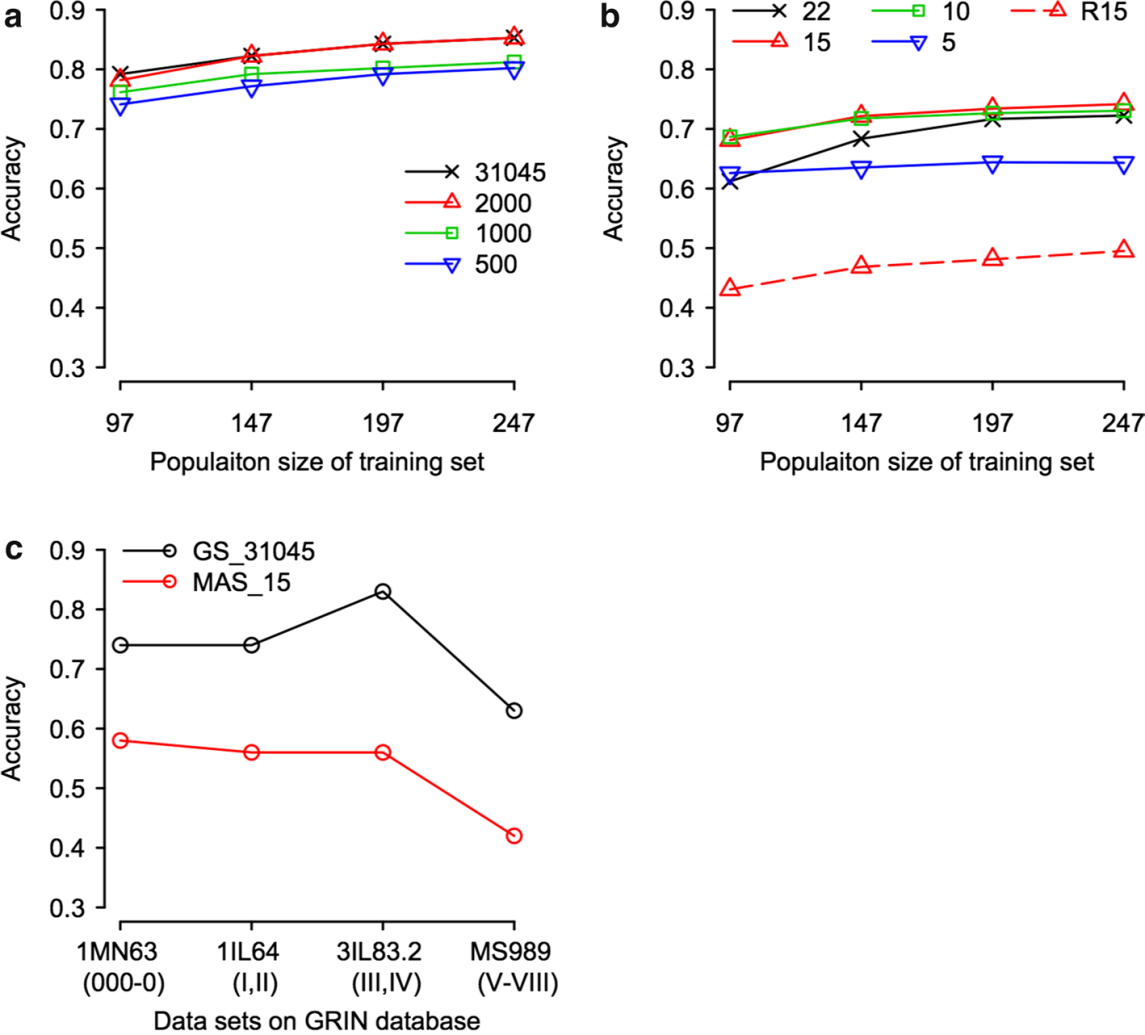
Prediction accuracies of genomic selection (*GS*) and marker-assisted selection (*MAS*) for the association panel and the panels obtained from GRIN. **a**, **b** The average prediction accuracies of 1000 iterations of GS and MAS for seed weight, respectively. The number of SNPs used for prediction was indicated in the legend. For GS with a subgroup of SNPs, an equal number of SNPs were randomly selected from each chromosome. For MAS, the prediction accuracies with 15 randomly selected SNPs (R15) were also plotted as a control. **c** The prediction accuracies of GS with the entire set of SNPs and MAS with the 15 selected trait-associated SNPs for seed weight of the four GRIN panels. The maturities of individuals involved in each panel were indicated in *parenthesis*

The prediction accuracies of GS and MAS using the loci identified via GWAS for SW were then investigated. When the training population reaches its maximum size (247), the prediction accuracies ranged 0.80–0.85 and 0.64–0.74 for GS and MAS, respectively, varying with the number of SNPs used for prediction (Fig. 4a, b). For GS, the prediction accuracy using 2000 SNPs was 0.85, similar to that with the whole set of SNPs. The prediction accuracy remained as high as 0.80 when the number of SNPs used for prediction decreased to 500. For MAS, as shown in Table 2, all 22 loci identified via GWAS and 5, 10 and 15 loci selected out of them were used, respectively. The highest prediction accuracy was 0.74 with 15 selected loci, which was 25 % higher than that with 15 random SNPs (Fig. 4b). The prediction accuracy decreased to 0.64 when only five selected loci were used. We also investigated the effects of training population size on the prediction accuracy of GS and MAS when a consistent number of 62 accessions were randomly assigned as validation set. As shown in Fig. 4a, b, the prediction accuracies for both GS and MAS decreased along with the reduction of the training population size, regardless of the number of loci used. The allelic segregations of the 15 selected loci in the five PIs with the extreme SW are presented in Table 3.

**Table 2:** Lead SNPs of loci selected by stepwise method for MAS based on Akaike information criterion

No. of SNPs	Selected SNPs for genetic breeding values prediction
5	Gm04_14213918, Gm14_5352488, Gm18_59490788, Gm19_41013395, Gm20_481573
10	Gm04_14213918, Gm06_13151347, Gm09_40164588, Gm10_37088544, Gm14_5352488, Gm18_59490788, Gm19_41013395, Gm19_41144271, Gm19_42921997, Gm20_481573
15	Gm04_14213918, Gm04_47414790, Gm06_13151347, Gm06_15115808, Gm07_15662403, Gm09_40164588, Gm10_37088544, Gm14_5352488, Gm14_6324126, Gm18_59490788, Gm18_61540919, Gm19_41013395, Gm19_41144271, Gm19_42921997, Gm20_481573

**Table 3:**
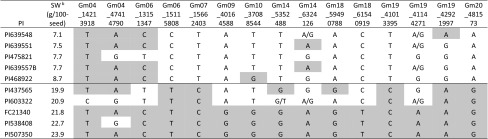
Allelic segregation of the 15 selected loci in the five plant introductions (PIs) with extreme seed weight (SW) in the association panel^a^

^a^The allele with positive effect on each locus is highlighted in red

^b^Shown are the averages over 4 environments and 3 replications for each environment

We further investigated the efficiency of GS with the entire set of SNPs and MAS with the 15 selected trait-associated SNPs, with which the highest prediction accuracy was realized in MAS as described above, in predicting SW using four unrelated populations obtained from GRIN (http://www.ars-grin.gov/). These panels represent a wide range of maturities (MG 000-VIII) and a large variation for SW (Supplementary file Table S1 and Fig. S5). An approximately normal distribution was observed for SW in the panels except MS989 with a later maturity (Supplementary file Fig. S5). The prediction accuracies of GS ranged from 0.63 for MS989 to 0.83 for 3IL83.2, 21 % averagely higher than that of MAS, which ranged from 0.42 for MS989 to 0.58 for 1MN63 (Fig. 4c).

## Discussion

In association study, relative kinship and population structure are two major confounding factors that may lead to spurious results (Yu et al. 2006). The MLM taking both familial relatedness and population structure into account has been demonstrated as an effective way to control genomic inflation and has been widely used in GWAS for various complex traits and plant species (Huang et al. 2010; Li et al. 2013; Morris et al. 2013). Our previous study also showed that a marker-based principal analysis is sufficiently flexible to generate trait-specific population structure that optimizes the model fitness (Zhang et al. 2015). In the present study, the first four PCs as suggested by BIC test were used to rule out spurious association due to population structure during association analyses (Supplementary file Table S3). We also compared different models and found that genomic inflation was attributed to both population structure and relative kinship in this study (Supplementary file Fig. S4). The MLMs (regular MLM and compressed MLM) showed a better control of genomic inflation than the naive model and the GLM with population structure, while the performances of the regular MLM and compressed MLM were similar (Supplementary file Fig. S4). It indicates that the feature of a trait should be taken into account for considering the optimal model, though compressed MLM was more powerful and effective in some association studies (Huang et al. 2010; Zhang et al. 2010).

In soybean, more than 200 QTLs for SW have been reported across 20 chromosomes (SoyBase, http://www.soybase.org/). Additionally, many QTLs associated with seed size, a trait highly related to SW, have also been identified. These QTLs could be used to confirm the loci identified via GWAS, especially the hot ones. In this study, 22 loci associated with SW were identified. Each individual locus could explain a small proportion (<4 %) of phenotypic variance. The result demonstrated that soybean seed weight is a typical quantitative trait, genetically conditioned by many minor-effect loci. Of the identified loci, 15 have been previously reported at least once (Table 1). Among these loci, the SW11 locus at 37.1 Mb position on Gm10 exhibited the strongest association. In the similar region, four SW QTLs have been reported previously. Another locus SW2 at the 14.2 Mb position on Gm04 has been reported to be associated with SW and seed size related traits, e.g. seed length, height and width, two and three times, respectively (Table 1). The SW21 on Gm19 was mapped within an overlapped region of six SW QTLs and six QTLs for seed size related traits reported previously, indicating that SW21 might be in one of the hottest regions related to SW in soybean (Table 1). The high repeatability of these loci across various environments and genetic backgrounds implies a great potential of marker-based breeding for SW in soybean. As SW plays an important role in soybean yielding, these loci are also useful for soybean yield improvement.

One of the primary advantages of GWAS is the high mapping resolution as compared with linkage mapping. This feature enables GWAS to further narrow down the chromosomal region of candidate QTLs and predict causal genes. In this study, we were able to map SW11 and SW22 to a chromosomal region of 218 kb on Gm10 and 65 kb on Gm20, respectively. Within the regions of SW11 and SW22, there were 13 and six putative genes located, respectively (Fig. 4). According to the “Seed development transcript count” track at the SoyBase website (http://soybase.org/), we further decreased the number of candidate genes to eight for SW11 and five for SW22, respectively. The closest seed transcript to the lead SNP of the SW11 locus, *Glyma10g28250*, was predicted to encode a MYB61-like transcription factor. In *Arabidopsis*, MYB61 is required for mucilage deposition and extrusion during imbibition of seed coat (Penfield et al. 2001). In mature seed, mucilage is present in a dehydrate form within epidermal testa cells, and is also important for seed germination and dormancy. Deficiency of mucilage was observed in some *Arabidopsis* mutants with phenotype in seed size and/or weight such as *ap2* and *transparenttestaglabra* (*ttg*) (Debeaujon et al. 2000).

In addition, some candidates of interest for other loci were also identified. The candidate gene for SW19 locus, *Glyma19g33421*, annotated as an AHP family member (Table 1; Fig. 2b). AHPs play a key role in the two-component pathway by which cytokinin signaling is conducted. In the two-component system, AHPs transmit cytokinin signal from the cytokinin receptors AHKs (also known as histidine kinase) to downstream *Arabidopsis* response regulators (ARRs) as well as cytokinin response factors (CRFs), which include a sub set of AP2 transcriptional factors (Rashotte et al. 2006). Both the triple mutant *ahk2,3,4* and the *ap2* mutant showed enlarged seed size mainly due to increased size of embryo (Ohto et al. 2005; Riefler et al. 2006). Interestingly, the *Arabidopsis* penta mutant *ahp1,2,3,4,5* has a similar phenotype of enlarged embryo and seed size (http://arabidopsis.org). Therefore, it is conceivable that *Glyma19g33421*, encoding an AHP protein, might be involved in cytokinin-mediated seed mass regulation pathway in soybean. The SW21 locus was mapped to a region on Gm19 similar to 12 previously reported QTLs for SW or seed size related traits. The candidate gene, *Glyma19g35180*, putatively encodes an AUX/IAA family protein (Fig. 2c). AUX/IAA family is one of the key regulators of the auxin-modulated genes and is involved in various developmental responses to auxin. It regulates the expression of auxin-induced genes by heterodimerization with ARFs (Reed 2001). *Arabidopsis* ARF2 is a general repressor of cell division. Loss-of-function mutant *arf2* showed extra cell division in the integuments and dramatically increased seed size and weight (Schruff et al. 2006). However, it is uncertain whether *Glyma19g35180* is involved in *ARF2* mediated SW signaling in soybean. More studies like functional analyses of candidate genes are required to validate their possible roles in determining soybean SW.

GS is based on the genetic effects of dense markers across entire genome (Meuwissen et al. 2001). It hypothetically captures all genetic variations of a trait, while MAS is usually based upon a small number of loci, particularly major-effect loci. In our study, GS consistently outperformed MAS for SW in soybean for various cross-validations within the association panel, even though in the case that the prediction accuracy of MAS might have been overestimated when the same population as for trait-associated loci identification was used. Moreover, the superiority of GS was more obvious when unrelated panels were used in validation (Fig. 4c), indicating that GS is more robust than MAS in predicting soybean SW. In addition to the reason mentioned above that more genetic variants can be captured by GS than by MAS, the higher prediction accuracy of GS can be partially due to its ability to well capture the genetic relationship between training and validation sets, which also contributes to the prediction accuracy (Habier et al. 2007).

Compared with a previous GS study in soybean, the prediction accuracy of GS in the present study was relatively high. Jarquin et al. (2014) conducted GS for grain yield, plant height and maturity date in a population of 301 elite soybean breeding lines with 52,349 SNPs. The highest estimate of broad-sense heritability was 0.94 for maturity date, which is similar to the SW (0.97) in this study. In that study, however, the highest heritability-adjusted prediction accuracy was 0.70 for maturity date with the optimal prediction model, which is lower than the highest prediction accuracy of 0.83 in the present study. Because the two studies had similar population size, number of SNPs and trait heritability, the difference in genetic architecture of traits and populations under study might be the major factors leading to the difference in prediction accuracies between the two studies.

Consistently with previous studies (Asoro et al. 2011; Jarquin et al. 2014), we found that the prediction accuracy increased as the training population size increased in both GS and MAS. However, this tendency appeared in different patterns. In GS, the effect of training population size was relatively stable over predictions with different number of SNPs (Fig. 4a). While in MAS, the effect of population size was magnified with the increase of loci used for prediction (Fig. 4b). It implies that the prediction of MAS is more sensitive to the change of training population sizes, which may lead to changes of relatedness between training and validation sets. Minimizing markers while keeping reasonable prediction accuracy is helpful to reduce genotyping cost in genomic prediction. We found that decreasing the number of SNPs did not necessarily sacrifice the prediction accuracies of GS, suggesting over-abundance of SNPs in the association panel. It can be explained by the extensive LD of the soybean genome and/or that not all SNPs contribute to the genetic variation of SW in soybean. Our results also suggested that a subset of 2000 SNPs (100 SNPs per chromosome) could be capable of providing equivalent prediction accuracy as the whole set of 31,045 SNPs did. Further decreasing the number of SNPs might be practicable, depending on the budge availability and the requirement for prediction accuracy. To the unrelated population, further work is needed to address the marker density effect and the over-abundance of SNPs in soybean GS. In addition, the prediction accuracy of MAS with all the 22 loci identified was lower than that with 15 or 10 loci selected. It suggested that more loci involved in MAS might not necessarily lead to higher efficiency as expected. Increasing the number of loci/markers used in MAS may not be an appropriate option in some cases even if efficiency of selection is the only issue to be considered.

In most of the GS or MAS studies, the training and validation populations are related, and cross-validation is used to estimate the prediction accuracy (Bao et al. 2014; Hoeck et al. 2003; Jarquin et al. 2014). However, their selection efficiency for unrelated lines is usually undetermined. In the present study, besides cross-validation, validation with unrelated populations obtained from GRIN (http://www.ars-grin.gov/) was also carried out. We found that the prediction accuracy of GS varied among the panels with different groups of maturity (Fig. 4c). This phenomenon might be caused by the different degrees of similarity in genetic compositions between the training panel and the validation sets. We noted that the prediction accuracies (0.74–0.83) for the early and medium maturity validation panels (MG 000-IV) were higher than that (0.63) of the late maturity panel (MG V-VIII). The high prediction accuracy of GS across a wide range of maturities may be benefitted from the broad genetic diversity of the association population and the dense SNPs used in the study. The latter helps to maximize the LD between markers and causal genetic variants and also increases the potential of capturing the genetic relatedness between training and test sets (Gowda et al. 2014; Habier et al. 2007). According to the information from USDA (http://www.usda.gov/), soybean cultivars with MG 000-IV accounted for over three-fourths of the U.S. soybean production in 2013. Therefore, GS with the marker effects estimated in this study hold a great potential in predicting soybean SW. Given SW is an important component of grain yield, the results are also useful for the genomic prediction and genetic improvement of soybean yield.

## Conclusions

In this study, we identified 22 loci associated with SW via GWAS and thus convincingly demonstrated that soybean SW is conditioned by numerous loci of minor effect. We also refined candidate chromosomal regions for the known QTLs, including the hotspots on Gm04 and Gm19. Candidate genes with *Arabidopsis* homologs involved in seed development, auxin and cytokinin signalings controlling SW were proposed. Cross-validation showed that MAS could be a competitive alternative of GS when training and validation population were related or had similar genetic components. However, validation with unrelated populations across a broad range of maturities suggested that GS is superior to MAS. This study enhances our knowledge of the genetic basis of SW in soybean, and facilitates exploring the molecular mechanisms underlying the trait. It also benefits the genomic prediction of yield in soybean and suggests that GS would have a great potential in increasing genetic gains of soybean breeding.

### Author contribution statement

J.Z. and G.-L.J. designed the research experiments. Q.S. and P.B.C. designed SNPs and performed genotyping. J.Z. performed phenotyping and analyzed data. G.-L.J. designed the overall project. J.Z and G.-L.J. wrote the manuscript.

## Electronic supplementary material

Supplementary material 1 (DOCX 887 kb)
