# Supplementary material for: Genome-wide association study, genomic prediction and marker-assisted selection for seed weight in soybean (Glycinemax)
Source: Theor Appl Genet. 2015 Oct 30;129:117–30. doi: 10.1007/s00122-015-2614-x (PMC4703630; doi:10.1007/s00122-015-2614-x)
Supplement: Supplementary file 1 — Supplementary material 1 (DOCX 887 kb) [file 122_2015_2614_MOESM1_ESM.docx]

**Supplementary material**

**“Genome-wide association study and marker-based selection for seed weight in soybean (*Glycine* *max*)”**

***Theoretical and Applied Genetics***

Jiaoping Zhang, Qijain Song, Perry B Cregan, and Guo-Liang Jiang*

J. Zhang

Plant Science Department, South Dakota State University, Brookings, SD 57006, USA

Q. Song and P. B. Cregan

Soybean Genomics and Improvement Laboratory, US Department of Agriculture, Agricultural Research Service (USDA-ARS), 10300 Baltimore Ave, Beltsville, MD 20705, USA

G.-L. Jiang*

Agricultural Research Station, Virginia State University, Carter G. Woodson Ave, P.O. Box 9061, Petersburg. VA 23806

Corresponding author and Email address:

G.-L. Jiang: [gjiang@vsu.edu](mailto:gjiang@vsu.edu) or [gljiang99@yahoo.com](mailto:gljiang99@yahoo.com)

**Fig. S1 The frequency distribution of averaged 100-seed weigh of 309 soybean germplasm accessions over 4 environments with 3 replication for each environment.**


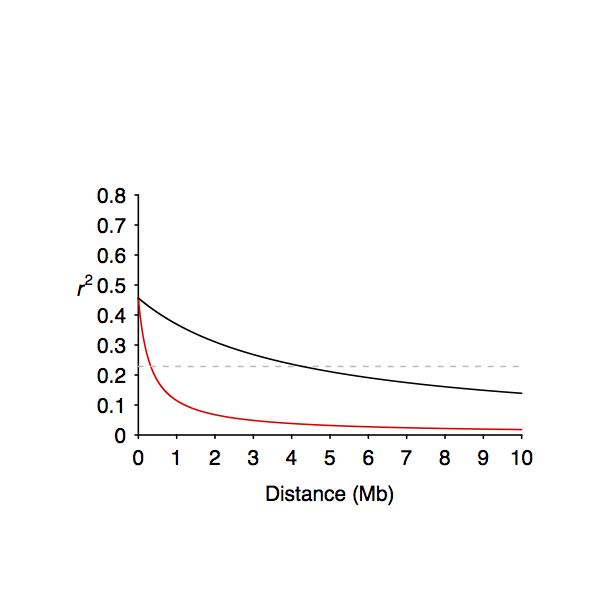


**Fig. S2 Average LD decay in euchromatic and heterochromatic chromosomal regions**.

The mean LD decay was estimated as squared correlation coefficient (*r*^2^) using all pairs of SNPs located within 10 Mb of physical distance in euchromatic (red) and heterochromatic (black) regions in a population of 309 soybean germplasm accessions. The dash line in grey indicates the position where *r*^2^ dropped to half of its maximum value.


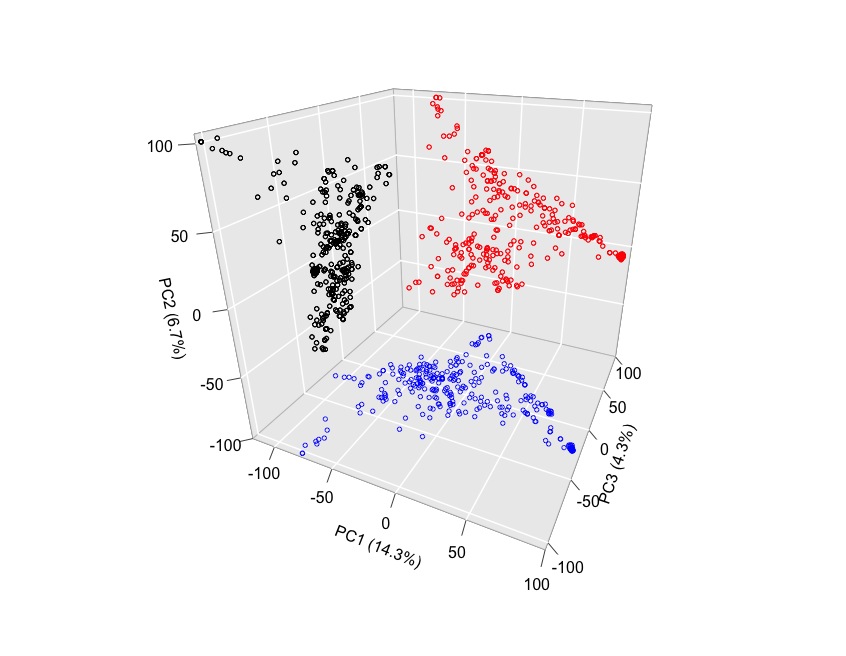


Fig. S3 Principal component (PC) analysis plot of the first three components of 309 accessions. Shown are the projection of the 3D plot of the first three PCs to the coordinators of PC1 and PC2 (red), PC1 and PC3 (blue), and PC2 and PC3 (black). The proportion of the total variance explained by each PC is given in the parenthesis.


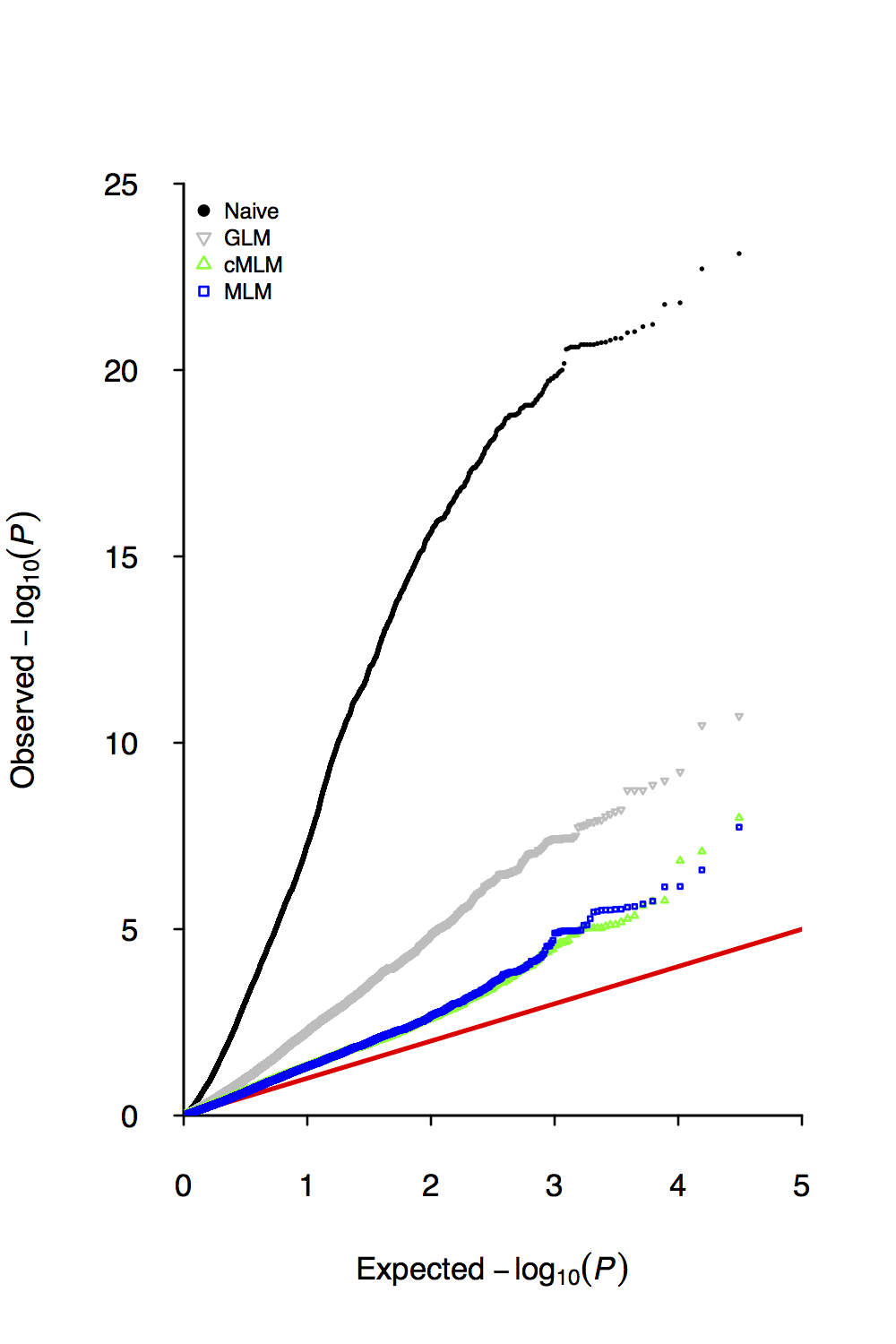


**Fig. S4 Quantile-quantile plot of association analysis for seed weight using different models.**

The observed *P-*values of naive model (without correction of population structure and kinship), general linear model (GLM) with correction of population structure, regular mixed linear model (MLM) and compressed MLM (CMLM) for 100-seed weight were plot against expected values. The expected uniform distribution of negative log_10_-transformed *P* values is indicated by the diagonal line in red. A statistic approach that has a distribution closer to the expected diagonal line indicates a better control of genomic inflation.

**Fig. S5 Frequency distribution of 100-seed weight of four GRIN panels.** a**.** Data of SOYBEAN.EVALUATION.1MN63 (MN63); b. SOYBEAN.EVALUATION.1IL64 (IL64). c. SOYBEAN.EVALUATION.3IL83.2 (IL83.2). d. SOYBEAN.EVALUATION.MS989 (MS989). The lines involved in the association panel of 309 PIs were excluded from analyses, and the final size of each population is given in the parenthesis.

| **Table S1 General description of the four GRIN panels.** | | | | |  |
| --- | --- | --- | --- | --- | --- |
| GRIN data set | Pop. Size^a^ | Maturity group | Mean ± SD | Range | |
| MN63 | 270 | 000-0 | 18.4 ± 4.4 | 4.0-33.8 | |
| IL64 | 724 | I, II | 15.2 ± 3.6 | 4.8-30.9 | |
| IL83.2 | 192 | III, IV | 14.4 ± 4.5 | 4.2-28.4 | |
| MS989 | 425 | V-VIII | 11.4 ± 3.9 | 4.0-29.0 | |
| ^a^ Population size after removing germplasm accessions involved in the association panel. | | | | |  |

| **Table S2 Analysis of variance of genetic effect (Gen), environmental effect (Env) and their interaction effect (Gen x Env).** | | | | |
| --- | --- | --- | --- | --- |
| Source | Df | SS | MS | *F* |
| Gen | 308 | 17134 | 55.7 | 38.0*** |
| Env | 3 | 5665.7 | 1888.6 | 73.8*** |
| Gen X Env | 923 | 1353.1 | 1.5 | 3.1*** |
| Block (Env) | 8 | 197.5 | 24.7 | 51.6*** |
| Residuals | 2455 | 1174 | 0.5 |  |
| *** *P* < 0.0001 | | | | |

| **Table S3 Bayesian Information Criterion (BIC) test of the fitness of model with different number of principal components (PCs).** | |
| --- | --- |
| Number of PCs | BIC^a^ |
| 0 | -531.79 |
| 1 | -534.65 |
| 2 | -532.22 |
| 3 | -524.76 |
| 4 | -523.43 |
| 5 | -526.29 |
| 6 | -527.93 |
| 7 | -528.35 |
| 8 | -527.98 |
| 9 | -530.44 |
| 10 | -533.30 |
| ^a^ Larger is better | |
